# The Effect of Cannabis-Based Medicine on Neuropathic Pain and Spasticity in Patients with Multiple Sclerosis and Spinal Cord Injury: Study Protocol of a National Multicenter Double-Blinded, Placebo-Controlled Trial

**DOI:** 10.3390/brainsci11091212

**Published:** 2021-09-14

**Authors:** Julie Schjødtz Hansen, Rikke Middelhede Hansen, Thor Petersen, Stefan Gustavsen, Annette Bang Oturai, Finn Sellebjerg, Eva Aggerholm Sædder, Helge Kasch, Peter Vestergaard Rasmussen, Nanna Brix Finnerup, Kristina Bacher Svendsen

**Affiliations:** 1 Department of Neurology, Aarhus University Hospital, DK-8200 Aarhus N, Denmark; petrasmu@rm.dk (P.V.R.); finnerup@clin.au.dk (N.B.F.); krissven@rm.dk (K.B.S.); 2 Department of Clinical Medicine, Aarhus University, DK-8200 Aarhus N, Denmark; helgkasc@rm.dk; 3 Spinal Cord Injury Centre of Western Denmark Viborg Regional Hospital, DK-8800 Viborg, Denmark; rikke.m.hansen@viborg.rm.dk; 4 Department of Neurology, Hospital of Southern Jutland and Research Unit in Neurology, Department of Regional Health Research, University of Southern Denmark, DK-5000 Odense, Denmark; Thor.Petersen@rsyd.dk; 5 Danish Multiple Sclerosis Center, Department of Neurology, Copenhagen University Hospital-Rigshospitalet, DK-2600 Glostrup, Denmark; stefan.gustavsen@regionh.dk (S.G.); annette.oturai@regionh.dk (A.B.O.); finn.thorup.sellebjerg@regionh.dk (F.S.); 6 Department of Clinical Pharmacology, Aarhus University Hospital, DK-8200 Aarhus N, Denmark; evasaedd@rm.dk; 7 Department of Neurology, Viborg Regional Hospital, DK-8800 Viborg, Denmark; 8 Danish Pain Research Centre, Department of Clinical Medicine, Aarhus University, DK-8200 Aarhus N, Denmark

**Keywords:** cannabinoids, THC, CBD, medical cannabis, cannabis-based medicine, multiple sclerosis, spinal cord injury, neuropathic pain, spasticity, numeric rating scale

## Abstract

Disease or acquired damage to the central nervous system frequently causes disabling spasticity and central neuropathic pain (NP), both of which are frequent in multiple sclerosis (MS) and spinal cord injury (SCI). Patients with MS and SCI often request treatment with cannabis-based medicine (CBM). However, knowledge about effects, side effects, choice of active cannabinoids (Δ9-tetrahydrocannabinol (THC), cannabidiol (CBD) alone or in combination), and doses of CBM remains limited. Using a double-blind, parallel design in a national multicenter cohort, this study examines the effect of CBM on spasticity and NP. Patients are randomized to treatment with capsules containing either THC, CBD, THC and CBD, or placebo. Primary endpoints are patient-reported pain and spasticity on a numerical rating scale. Other endpoints include quality of life and sleep, depression and anxiety, and relief of pain and spasticity. Side-effects of CBM are described. In a sub-study, the pharmacodynamics (PD) and pharmacokinetics (PK) of oral capsule CBM are examined. We expect that the study will contribute to the literature by providing information on the effects and side-effects of CBD, THC, and the combination of the two for central neuropathic pain and spasticity. Furthermore, we will describe the PD/PK of THC and CBD in a patient population.

## 1. Introduction

Disease or acquired damage to the central nervous system (CNS) may entail disabling spasticity and central neuropathic pain (NP) [1]. In patients diagnosed with multiple sclerosis (MS) or spinal cord injury (SCI), these symptoms are frequent and prominent.

MS causes multifocal and diffuse injuries to the CNS. Over time, most patients with MS (pwMS) develop significant symptoms with loss of function and a certain degree of disability. About 65% of pwMS report pain, and approximately 26% have central NP [2], which significantly impacts their quality of life [3]. About 2/3 of pwMS suffer from spasticity (tightness/stiffness of the muscles) [4] and 15% of pwMS report spasm-related pain [2]. Spasticity and spasms often have a significant impact on sleep [5] and everyday life, and are limiting factors in rehabilitation. 

The consequences of SCI depend on the neurological level and the extent of the injury. Approximately 50% of patients with SCI (pwSCI) report significant chronic central or peripheral NP [6]. In a recent Danish questionnaire survey in pwSCI, 71% reported spasticity and 46% received medical treatment for spasticity [7]. No curative treatment exists for SCI. Hence, a good rehabilitation offer including optimal symptomatic treatment to prevent long-term complications is important [8]. 

Traditionally, spasticity has been treated with baclofen or tizanidine, but the effects of these pharmaceuticals are often not sufficiently felt by many patients at the maximum dose [9], or they discontinue treatment due to side-effects. 

Treating NP is a challenge since the available analgesics often only have partial or no effect [10], and new treatment regimens, such as medical cannabis, are therefore being sought.

For centuries, cannabis has been used for various purposes [11]. Lately, interest in cannabis, cannabinoids, and cannabis-based medicines (CBM) for medical purposes has been growing [11,12]. CBM has been suggested as a treatment for a wide range of diseases and symptoms. For research purposes, the potential benefit of CBM for spasms, pain, and sleep disturbance are of certain interest [13,14]. Many pwMS and pwSCI have tried CBM to relieve their symptoms, or they demand treatment with CBM [15,16,17,18,19]. 

CBM is based on cannabinoids originating from the *Cannabis sativa* plant, which has more than 120 cannabinoids [20]. These cannabinoids can be produced as either natural (plant-derived) or synthetic compounds. The predominant effect of cannabinoids in humans is exercised via the cannabinoid receptor type 1 (CB_1_) and cannabinoid receptor type 2 (CB_2_) [20,21]. The most potent psychoactive cannabinoid is Δ9-tetrahydrocannabinol (THC) [22]. THC binds to the CB_1_ receptor and acts as a (partial) agonist. CB_1_ receptors are primarily found in the presynaptic part of the nervous system [11,23]. Cannabidiol (CBD) is another important cannabinoid. CBD allosterically blocks the CB_1_- and CB_2_ receptor (with low affinity) and therefore has antagonistic effects compared with THC (e.g., downsizes some of the (side-)effects of THC) [22,24,25,26]. CB_2_ receptors are primarily but not exclusively expressed in immune cells [21]. In animal studies, combination treatment (THC:CBD) is superior to THC and CBD alone [27], but the extent to which it also applies to humans is uncertain.

Whole-plant extracts have been suggested to have a better therapeutic effect than synthetic CBM because of entourage effects with terpenoids [25]. However, Bon-Miller et al. argued that the entourage theory has certain limitations because it is difficult to demonstrate and it is not clear, which compound drives the effect. Furthermore, they address the misconception that natural cannabinoids are superior to synthetic cannabinoids at the single molecule level [28]. When purifying cannabinoids from currently available pure chemotypes (whole-plant extract), it is noteworthy that CBD-predominant types all have the presence of THC (4–5% of the cannabinoid fraction) [11]; and at the time of trial approval, no plant-based CBD product fulfilled the authorities’ requirements for use in clinical trials. On the other hand, THC-predominant chemotype plants contain practically no CBD [11]. Hence, purified THC can be plant-based. Previously, these manufacturing issues were rarely addressed.

The effects and pharmacokinetics (PK) of CBM depend on the formulation and route of administration [29]. Inhalation of cannabinoids reaches a peak plasma concentration within 3–10 min. Oral formulation undergoes hepatic first-pass metabolism and its absorption can vary. Hence, the peak plasma concentration is lower with oral formulation than with inhalation and has a delay (~120 min). The potential benefits of oral formulation are symptomatic relief over a longer period (steadier plasma concentration over time) and the ease of use (to which the certainty of dose, preparation, and storage solutions should be added). However, the description of the pharmacodynamics (PD) and PK of the oral capsule CBM in a patient population is lacking [29]. 

In the past two decades, numerous studies have been conducted to determine the effects of CBM.

Previous studies on the effect of CBM for NP and spasticity have shown limited and conflicting evidence. Among these studies, those that investigated the effect of cannabis (smoked) or CBM on spasticity in pwMS have indicated a small positive effect [30,31,32,33,34,35,36]. In Denmark and several other countries, the oromucosal spray nabiximols (a cannabis extract product containing CBD and THC) has been approved as an add-on therapy for spasticity for pwMS if other treatments are ineffective [37]. Minor studies have been conducted investigating CBM for pwSCI which have shown some effect on spasticity, but sample sizes have been small [38,39]. 

Studies examining CBM for NP report divergent results. Only a few studies found a positive effect of CBM on neuropathic pain [40,41], but most studies investigating nabiximols report negative outcomes [10]. Several reviews and meta-reviews conducted on the topic of CBM and NP all conclude that there is no or very limited effect of CBM [13,42,43,44,45]. The most recent recommendation (2021) from ‘The International Association for the Study of Pain’ (IASP) does not endorse the use of cannabis and cannabinoids for treating pain due to lack of high-quality clinical evidence [46]. In summary, at this point it is difficult to determine unequivocally whether CBM has a place in the treatment regime for NP and spasticity in pwMS or pwSCI. 

The side-effects of CBM are important to acknowledge when discussing its potential use for symptomatic treatment. Of special concern is the risk of (long-term) psychiatric disease, risk of addiction, affected memory and driving skills (response retardation) [14,47,48]. Nonetheless, the adverse event (AE) profile of CBM is poorly understood. It is noteworthy that studies investigating isolated CBD for NP and spasticity are almost non-existent and that the exact interaction between CBD and THC is poorly understood. 

The aim of this study is to investigate the effects of CBD, THC, and the combination of the two on NP and spasticity in pwMS and pwSCI over a 6-week treatment period. A comprehensive description of side-effects is performed; and in a sub-study, the PK and PD of CBM capsules are examined. We expect that the study will shed further light on possible effects and immediate side-effects of THC, CBD, and their combination.

## 2. Materials and Methods

### 2.1. Study Design and Settings

The study is designed as a national multicenter randomized double-blind, placebo-controlled parallel study (RCT). Patients are randomized to one of four treatment arms: THC, CBD, THC and CBD, or placebo.

**Figure 1:**
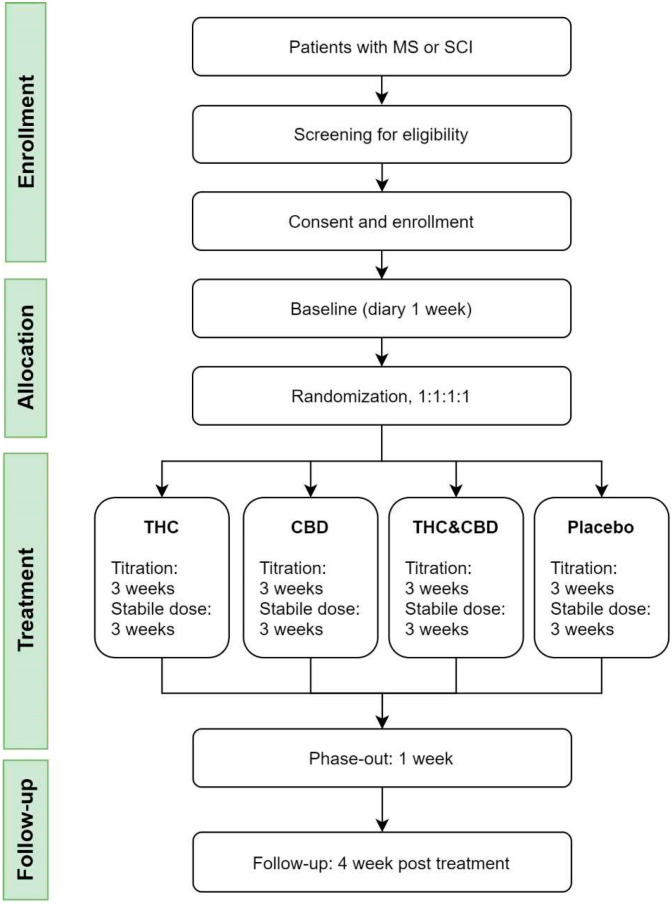
Flow diagram.

The study is coordinated by the steering committee with representatives from the Dept. of Neurology, Aarhus University Hospital (AUH); the Spinal Cord Injury Centre of Western Denmark (SCIWDK); the Danish Pain Research Center; the Dept. of Clinical Medicine, Aarhus University; the Danish Multiple Sclerosis Centre; the Dept. of Neurology, Copenhagen University Hospital-Rigshospitalet (DMSC); and the Dept. of Clinical Pharmacology, AUH. The Danish MS clinics and the two national SCI clinics participate in the study. The study plans to include 448 patients for the main study and 40 patients for the sub-study. All patients are randomized to one of four treatment arms (Figure 1). THC is naturally extracted from the *cannabis sativa* plant. CBD is synthetically produced. Maximum daily doses are THC = 22.5 mg and CBD = 45 mg. 

The treatment duration is 7 weeks in total, with endpoints evaluated at the end of the sixth week. Previous studies suggested an effect after 4–5 weeks of treatment [34,49] Standard Protocol Items: The Consolidated Standards of Reporting Trials (CONSORT) 2010 checklist and flow diagram will be used when presenting the results of the present study.

### 2.2. Study Subjects

PwMS Affiliated with or referred to one of the MS Clinics (at Neurological Departments) in Denmark. PwSCI Affiliated with or referred to one of the two National Clinics for Spinal Cord Injury in Denmark.

Eligible patients are informed about the study and recruited at the scheduled outpatient controls in the clinics. Additionally, the patient organizations for MS and SCI advertised the study on their webpages and member forums. In the case of interest in participation, participant information will be forwarded. 

Patients are screened for eligibility. Informed consent is obtained. For participation, the patient’s full medical history, clinical and neurological examination results, echocardiogram, and biochemistry will be obtained. Patients who, after a 7-day baseline period, meet all inclusion criteria and no exclusion criteria are randomized for treatment (Table 1).

A screening log is kept. Screening, inclusion, and randomization are performed by specialists in neurology or spinal cord injury at resident level or higher. We expect that more pwMS than pwSCI will be included.

#### 2.2.1. The Main Study

After 7 days of baseline registration, the treatment dose titration phase begins according to a standardized 3-week schedule. If tolerated, the dose is increased to the maximum (minor doses can be used in case of non-tolerable side-effects or if assessed by the patient to have sufficient effect on NP and/or spasticity). After the dose titration phase, the treatment is continued for 3 weeks at a steady dose, after which the drug is phased out over 1 week. A follow-up telephone consultation is performed 4 weeks after treatment. The patient keeps a diary during the entire medical trial. The patients record the average intensity on a numeric rating scale (NRS 0–10) for pain/spasticity and sleep, the use of paracetamol, change in medication, predefined titration plan, and how many capsules (project medication) they have taken per 24 h. Weekly during the dose titration phase, there will be telephone consultations. Other medications for spasticity and/or pain are continued at a stable dose. Concomitant therapies (including rehabilitation, physiotherapy, occupational therapy, splints and orthoses, etc.) and medication (including spasmolytics and analgesics (except opioids)) are maintained during the course of the study. Paracetamol is allowed as escape medicine. If a patient discontinues treatment before the scheduled time, the investigator will collect information on the cause, side-effects, and effect at the time of cessation. The patient is asked to continue the diary and participate in the medical check at week 6. If this is not possible or the patient wished to withdraw from the project, a follow-up telephone consultation will be agreed 2 weeks after the withdrawal. The patient is monitored for any AEs due to the project medicine. 

#### 2.2.2. The Sub-Study

PK and PD are investigated in a subset of 40 patients from the main study, all recruited from AUH. The patients will be admitted to the hospital for a 24 h period while in a steady state with the project medicine (preferably week 6). During the 24 h period, pain (NRS 0–10) and side-effects are registered and blood samples collected for PK/PD (blood samples are taken before the first dose on the startup day and then at 1 h, 2 h, 4 h, 6 h, 8 h, 10 h, 12 h, 15 h, 18 h, 21 h, and 24 h after the first dose). The blood samples will be kept at −80 degrees until analysis. Blood tests are analyzed at the Institute of Legal Medicine, Dept. of Forensic Medicine, Aarhus University, where full blood will be analyzed for THC, cannabinol (CBN), and CBD; and the metabolites 11-hydroxy-Δ9-tetrahydrocannabinol (THC-OH), and 11-nor-9-carboxy-Δ9-tetrahydrocannabinol (THC-COOH) by high-throughput liquid chromatography-mass spectrometry, LC-MS-MS [51].

### 2.3. Study Visits

Patients are scheduled to five visits at the clinic during the study. Telephone consultations are made during the titration phase, at follow-up, and by need. The electronic diary (e-diary managed by REDCap^®^) is completed during baseline and during the study treatment phase. Echocardiogram, biochemistry, medical examination, clinical tests, and so forth are performed during the study, as illustrated in Table 2.

### 2.4. Randomization and Blinding

Randomization is performed centrally at Glostrup Pharmacy using a computer-generated randomization list. Patients are randomized to the project after baseline if they are eligible. Block randomization is used at three sites (AUH, DMSC, and SCIWDK) and for the sub-study to ensure an approximately equal number in each treatment arm. No stratification is performed. The study is double-blind (patients, health care providers, and investigators/outcome adjudicators). Randomization codes are stored at the pharmacy until the project is completed. Randomization for the individual patient will only be broken in the case of serious adverse events (SAE), serious adverse reactions (SAR), or suspected unexpected serious adverse reactions (SUSAR). If the code is broken for an individual patient due to SAE, SAR, or SUSAR, only the study sponsor will know to which treatment arm they belong. Hence, treatment will still be blinded for investigators and statisticians. 

### 2.5. Intervention

The project medicine contains cannabinoids/placebo and is manufactured and packaged at Glostrup Pharmacy. Both the placebo and the three active treatments are given as an oral treatment in capsules.

One capsule contains: Hard gelatin capsule, white size 1 (HGC SIZE 1, Capsule). The empty capsules consisted of 2% titanium dioxide (Ph, Eur), qsq. 100% Gelatin (Ph.Eur) Excipients: Ascorbyl palmitate Ph.Eur. 0.2 mg, Hard Fat Ph.EUR Ad 0.5 mL, as well as an active component in the form of either:

(1) Dronabinol (THC): Dronabinol natural DAC 2.5 mg (maximum daily dose 22.5 mg); or
(2) Cannabidiol (CBD): Cannabidiol, synthetic DAC 5 mg or (maximum daily dose 45 mg); or
(3) Dronabinol (THC) + Cannabidiol (CBD): Dronabinol natural DAC 2.5 mg and Cannabidiol, synthetic DAC 5 mg (maximum daily dose 22.5 mg THC and 45 mg CBD); or
(4) Placebo: No active components.

The capsules are identical without smell, taste, color, or other characteristics that could lead to unblinding.

The selected maximum doses of the project medicine have been chosen to ensure that all patients reach an adequate dose to cover symptoms. The slow up-titration ensures that the best-tolerated dose is reached for each patient [52].

### 2.6. Outcome Measures 

Primary endpoint evaluation (main study):

(1) Pain intensity (PI): Average pain score in the diary during the past 7 days of active treatment at a stable dose (week 6) compared with the 7-day baseline period (NRS 0–10, where 0 is no pain and 10 is the worst possible pain).
(2) Degree of spasticity: Average spasticity score in the diary during the past 7 days of active treatment at a stable dose (week 6) compared with the 7-day baseline period (NRS 0–10, where 0 is no spasticity and 10 is the worst possible spasticity).

We hypothesized that there is no difference (0-hypothesis) between active treatment and the placebo when it comes to the primary endpoints (NP and spasticity) or adverse reactions. 

Secondary endpoint evaluation (main study):

(1) Patient Global Impression of Change (PGIC, 7-point scale from greatly worsened to greatly improved)
(2) Quality of Life (EQ-5D) [53]

Other endpoints

(1) Number of responders with 50% pain reduction/50% reduction of spasticity (diary)
(2) Pain relief and relief of spasticity (complete, good, moderate, mild, no, worsening, diary)
(3) The effect on various pain symptoms (NPSI) [54]
(4) Use of escape medicine (paracetamol, diary)
(5) Spasticity assessed on the Modified Ashworth Scale [55]
(6) The influence of pain and spasticity on activities, mood, and sleep (NRS 0–10, diary)
(7) Sleep (rated with NRS; 0: No sleep problems; 10: Worst possible sleep problems, diary)
(8) Sleep (PROMIS, diary) [56]
(9) Stress (PASAT, questionnaire) [57]
(10) Cognition (SDMT, Symbol Digit Modalities Test) [58]
(11) Coordination (MS), 9-hole peg test [59]
(12) Anxiety and depression (PROMIS, diary) [60]
(13) Patient’s expectation of pain relief (diary) (predictor)
(14) Trail Making Test A and B [61]
(15) Blinding (the patient must indicate what he/she thinks he/she has received at week 3 and 6)
(16) Side-effects (e-diary and visits) (list; dry mouth, headache, depression, nightmare, euphoria, dizziness, tinnitus, anxiety, hallucinations, fatigue/drowsiness, palpitations, flushing of the face, stomach ache, nausea, diarrhea, muscle pain, visual disturbances, and other (open question)).

Sub-study:

(1) PK and PD (maximum plasma concentration (Cmax), minimum plasma concentration (Cmin), average plasma concentration (Cavg), mean steady-state area under the curve (AUC_0-24_), Tmax, Tmin).
(2) The pharmacodynamic parameters: Pain intensity (PI), pain intensity difference (PID), pain relief, patient and investigator global evaluation.
(3) Pain registration on a NRS 0–10
(4) At the same time as the pain assessment, general questions are answered on a NRS of 0–10.

### 2.7. Statistics

For primary outcomes, the mean value (NRS) of the patients’ 7-day registration before treatment (baseline) will be compared to data from the last 7 days of active treatment at a stable dose (week 6). If a patient fulfills the inclusion criteria for both pain and spasticity, both endpoints will be evaluated. If only one of the effect parameters is present at inclusion (pain or spasticity), only the corresponding effect parameter will be evaluated as a primary endpoint. A comparison of the results from Visits 2 and 4 will be made for secondary and other outcomes (an exception is that pain outcomes are not evaluated if patients report no pain at inclusion, and vice versa for spasticity outcomes). Side-effects are collected as reported (diary, telephone consultation, or visits). By comparison of mean values (NRS), differences between treatment arms are analyzed with the one-way analysis of variance or Kruskal-Wallis test (with non-standardized data). For nominal data, differences are analyzed between groups with the chi-square test; and by data from the ordinal scale, differences between groups are analyzed with the Kruskal-Wallis test. We will adjust for the covariates of sex and age.

Pharmacokinetics/dynamics: Simple linear regression is performed on C_max_, C_min_, C_avg,_ and AUC_0-24_ as a function of a cannabinoid dose to investigate the linearity of cannabinoids. For the area under the curve of PI and PID (Proportional (P), Integral (I), and Derivative (D)), are summarized using standard methods. In the case of patient withdrawal from the trial for one reason or another, data collected during their participation will be included in the analysis (intention to treat analysis). The last week’s treatment will be used as endpoint (last observation carried forward (LOCF)). Other missing data are not replaced. If a large drop-out is observed before patients reach a steady state, analyses of the worst/best case scenario will be performed by a biostatistician.

### 2.8. Sample Size

For the main study, we calculated a standard deviation of 3.5 (SD3.5), an effect of 1 point on the NRS scale (difference from baseline mean NRS 5 to mean NRS 4 in treatment week 6), and a power of 80%. Considering four treatment arms and an uneven distribution in pain and spasticity, we expect that only about 40% of pwMS suffer from both pain and spasticity (opposite to about 70% in pwSCI). We aim to include 112 patients in each treatment arm (*n* = 448) (*n* = 480 when adjusting for drop-outs).

For the sub-study, the expected kinetics/dynamics are unknown. This is an exploratory study and no power calculation has been made.

### 2.9. Ethical Considerations and Safety

The study complies with the law on the processing of personal data. Trial participants receive both written and oral information before inclusion in the study and the voluntary nature of their participation will be emphasized. The patient may withdraw their informed consent for the project without justification at any time. Data from the project are stored electronically in REDCap^®^ (Aarhus University, Aarhus, Denmark), which can only be accessed by the project participants and monitors. REDCap^®^ is a secure web platform for building and managing online databases and surveys. Unexpected AEs are not expected, as the dose of THC and CBD in this study is at the same level or lower than in comparable studies [50]. In the study, patient safety is highly valued. Patients receive standardized treatment with a slow up-titration, and individual adjustment can be performed. Patients are associated with a single clinic, where they come for scheduled check-ups. All patients receive a personal contact card. All side-effects and AEs are documented. If an AEs are tolerable or unrelated to the study medication, treatment can continue with a close collaboration between the investigator/sponsor and the patient. If an AE is intolerable, patients are able to withdraw from treatment. SAE, SAR, and SUSAR are reported to the health authority within the timeframe set by Danish law, and a yearly safety report will be performed. Driving motorized vehicles is not permitted when on study medication. All women between menarche and post-menopause are defined as fertile, unless permanently sterile. Anticonception (as defined by the Danish Ministry of Health) should be used during and 3 months after treatment. Pregnancy tests will be performed for fertile women before enrollment and during the study.

Un-blinding will be performed after the last patient’s last visit (LPLV). If desired (stated at inclusion), study participants will receive a layman summary.

### 2.10. Publication

The results of the trial will be published in a recognized international journal regardless of the outcome (positive, negative, or inconclusive). The Vancouver recommendations for authorship will be followed. The results of the sub-study may be published independently.

## 3. Trial Status

The inclusion of patients was initiated in February 2019 and will continue until December 2021 at the latest.

## 4. Discussion

Previous studies have shown diverse results when it comes to CBM for treating NP and spasticity in pwMS and pwSCI. Recent reviews and recommendations all recommend more studies and evidence, as studies of the efficacy of CBD are especially lacking. The demand for CBM from patients and society is increasing, and treatment strategies are much sought-after.

The main aim of the present study is to investigate the effects and side-effects of CBM in pwMS and pwSCI. The study has numerous strengths. First, it is an investigator-initiated trial. Second, we use a relatively large sample size to reduce potential bias. We decided to investigate two patient groups with similar symptoms. It is plausible that their nerve damage is comparable, even though MS and SCI have different etiologies. Despite such potential differences in etiology, treatment of symptoms following nerve damage could be consistent [62,63]. Hence, we seize the opportunity to combine two patient groups in this study. Third, we have thoroughly conceived the study design. We investigate the two most widespread cannabinoids (THC and CBD) alone and in combination in a formulation where we know the exact amount of the administered drugs. By using capsule formulation, we limit the risk of unblinding. Fourth, we warrant an adequate dose of CBM in a secure setting with the possibility of individual adjustment. To avoid the need for imputation for the primary outcome, we ask all patients to continue to fill in their diaries even if they drop out of the study. Fifth, we have a proper setting for the PD/PK sub-study, investigating the metabolism of cannabinoids in a patient population. Finally, we make an extensive examination of side-effects.

The present study also has some limitations. It can be difficult to reach the desired number of participants. We have a strict set of exclusion criteria. Participants are not allowed to drive during the study, which can cause lower levels of engagement and a shift toward older and more advanced participants (as they may be less dependent on driving). The maximum dose of THC is in accordance with the doses in Sativex and dronabinol, and has been chosen to minimize the risk of side-effects. The maximum dose of CBD has been chosen to increase the possibility of effect. However, as no prior dose-response study has been performed, we may be at risk of either over- or under-dosing in this study. The availability of cannabinoids, especially CBD, has increased exponentially in recent years, and many patients already use CBD. Patients who experience an effect of CBD/CBM on their symptoms may be less willing to participate in the study, which can lead to underreporting of effects. The duration of treatment is limited to 7 weeks with a follow-up after 1 month. Therefore, we will not able to address the long-term effects or side-effects. The possibility of extension was discussed, but extension would lead to other issues (e.g., maintaining the blinding). There is a risk of unblinding due to the side-effects of CBM, but we will assess blinding success by asking the patients what treatment they think they have received.

The effect parameters in this study are primarily patient-reported outcome measurements (PROMS), which have been chosen to evaluate the patient’s perspective on the impact of treatment on their symptoms. However, the PROMS may have limitations because it is an individual and subjective outcome. We minimize the risk of recall bias by using a continuous daily diary during the study. To ensure a proper registration of side-effects, patients are asked predefined symptoms in a questionnaire, and they have the choice to report additional side-effects.

## Figures and Tables

**Table 1:** Inclusion and exclusion criteria.

	Inclusion Criteria
1	Definite or probable NP [1] for more than 3 months with mean pain intensity in baseline NRS > 3 and NRS ≤ 9 and/or presence of spasticity of more than 3 months with an intensity of >3 (NRS)
2	Stable disease (for pwMS; no relapse within the past month and no change in disease-modifying treatment during the previous three months).
3	Age ≥ 18 years
4	Informed consent is available.
	**Exclusion Criteria**
1	Competitive pain diseases (such as diabetic neuropathy) that cannot be distinguished from the patient’s pain due to SCI or MS
2	Opioid treatment that cannot be discontinued
3	Previous psychiatric disease in patient or nearest biological family, except well-treated depression
4	Previous risk of suicide assessed by the Columbia Suicide Severity Rating Scale [50]
5	Pregnancy and breast-feeding
6	Liver or renal insufficiency
7	Cardiovascular disease (except well-treated hypertension)
8	Previous convulsions/epilepsy
9	Active cancer disease
10	Previous or current addiction to alcohol/medication/drugs or positive urine screening
11	Current recreational cannabis use, or use within three months
12	Medical cannabis prescribed within 3 months
13	Allergy to cannabis products
14	Scheduled surgery during the study
15	Planned travels abroad during the study

**Table 2:** Standard Protocol Items.

	Inclusion	Baseline	Randomization	Titration			Stable Phase (Maximum Dose)				Phase Out		Follow-Up
Week	−4–0	0	Week 1 Day 1	1	2	3	4	5	6		7		11
VISIT	1		2			3			4		5		
Medical Examination	x		x							x			
Medical history	x												
Neurological exam.	x									x			
EDSS/ISNCSCI	x												
Heart and lung stethoscopy	x												
MAS	x									x			
Clinical tests													
9-hole peg test			x							x			
PASAT			x							x			
NPSI			x							x			
SDMT			x							x			
Trail making test			x							x			
EQ5D5L			x							x			
PGIC										x			
Others			x			x				x			
Pregnancy test			x			x							
Biochemistry *	x					x				x			
U-stix (Drug/THC)			x										
Electrocardiogram	x					x				x			
Blood pressure	x		x			x				x			
Expectation of relief			x										
Blinding **						x				x			
Dispensing of medicine			x			x							
Returning packaging						x				x		x	
Counting of capsules						x				x		x	
Admission ***									x				
Telephone				x	x	x							x
eDiary		x		x	x	x	x	x	x		x		
Pain (NRS)		x		x	x	x	x	x	x				
Spasticity (NRS)		x		x	x	x	x	x	x				
Relief pain (NRS)						x			x				
Relief spasticity NRS						x			x				
Sleep (NRS)		x		x	x	x	x	x	x				
PROMIS sleep		x				x			x				
PROMIS anxiety/depression		x				x			x				
Influence of pain/spasticity (NRS) -activity, mood and sleep		x				x			x				
Escape medicine				x	x	x	x	x	x		x		x
Side effects/AE				x	x	x	x	x	x		x		x

Legend Table 2: EDSS- Expanded disability status scale, ISNCSCI- International Standards for Neurological Classification of Spinal Cord Injury, MAS -Modified Ashworth Spasticity, PASAST- Paced Auditory Serial Addition Test, NPSI-Neuropathic Pain Symptom Inventory, SDMT-Symbol Digit Modalities test, 5Q5D5L Questionnaire 2009 EuroQol Group EQ-5D™(Danish), PGIC-Patient Global Impression of Change, NRS- Numeric rating scale. AE–Adverse events. * The biochemistry covers sodium, potassium, creatinine, eGFR, ALAT, urea, alkaline phosphatase, bilirubin, total cholesterol, low-density lipoprotein, high density lipoprotein, triglyceride, leukocytes, hemoglobin. INR if in anticoagulation therapy ** Patients are asked what kind of treatment they think they are having (and why). *** The admission for Sub-study II is in the stable phase (preferable in week 6, but 4 and 5 is accepted).
